# Peer Review of “Medical Brain Drain From Southeastern Europe: Using Digital Demography to Forecast Health Worker Emigration”

**DOI:** 10.2196/34079

**Published:** 2021-11-30

**Authors:** Monika Komušanac

**Affiliations:** 1 Department for Demography and Croatian Emigration Faculty of Croatian Studies University of Zagreb Zagreb Croatia

**Keywords:** digital demography, Google Trends, the emigration of doctors and nurses, medical brain drain, Croatia, demography, brain drain, emigration, doctors, nurses, health care workers, health professionals, health systems, jobs, Germany, personnel, migration, workforce, medical professionals

*This is a peer-review report submitted for the paper “Medical Brain Drain From Southeastern Europe: Using Digital Demography to Forecast Health Worker Emigration.”*

## Round 1 Review

### General Comments

This paper [1] represents an important contribution to the development of digital demographic methods and digital tools that can monitor and analyze demographic processes, especially migration as a highly dynamic component of population movement. This is extremely important in the management and planning of population development, especially in those populations that, as the examples of countries (subject areas) mentioned in the text, are struggling with intensive and increased emigration of young people. Given the lack of such studies, I consider this paper very important for developing new methods in demography and therefore suggest that you publish this paper because it represents an excellent starting point for further research and validation of digital traces that enrich the classical demographic methodological system and alleviate existing methodological limitations in the monitoring migration process.

This paper examines negative demographic processes and differences in the health system of countries of origin and destination with the example of the Google Trends tools combined with standard demographic analysis and official statistics to confirm the outflow of medical workers from the Western Balkans and Croatia to Germany and Austria.

The main goal of the work is to be satisfied and that is to determine the correlation between the official data and auxiliary data collected by the Google Trends tool. The results showed a high level of interdependence of trends shown by official statistics and analysis of Google Trends data by searching for relevant terms and keywords. The author pointed out the basic methodological limitations and advantages of using digital methods and tools with the intention to point out how the advanced digital age provides us a number of digital traces that can be used for observing demographic processes, especially migrations in the future.

### Major Comments

1. The lack of demographic potential in the health system is current and interesting, and the topic is actually a case report of the Western Balkans and Croatia through new approaches and tools of digital tracing and monitoring of migration processes. The author introduced readers to the demographic situation in the countries of origin to create a basis for understanding the processes that preceded the emigration of health workers based on a review of previous research and relevant authors.
2. The value of the paper is that the author, in addition to appropriate, standard, and common methods in demographic analysis, also used the method of tracking digital traces that is perhaps more common in other social sciences. He thus made an important step forward in methodological terms and focused on new approaches that will, in accordance with advanced and dynamic information technology, be more and more present and will provide us an important source of information and knowledge. Therefore, the development of digital methods in demography in the methodological sense is an expected necessity and an aid to the study of demographic processes and phenomena.
3. The results and the main findings of the paper are clear and unambiguous, and confirm the hypotheses, they are supported by data and graphical attachments, and they confirm the basic goal of the paper. The selected conclusions confirmed the importance of the new approaches and the connection that exists between the analysis of Google Trends and official data on the emigration of health workers to Germany and Austria using classical demographic methods.
4. The writing style is simple, clear, and understandable to the reader, and the issues are addressed clearly, concisely, and meaningfully. The research was conducted in accordance with ethical standards and the GDPR, and the data used by the Google Trends tool are anonymous and aggregate, and no one’s personal rights have been violated.
5. The paper shows a disproportion between the cited references and those in the bibliography; some references were mentioned in the text but are not listed in the bibliography and vice versa, some listed in the bibliography are not cited anywhere so the author is invited to revise bibliographic references and harmonize the text and the list of references.
